# Integrating hepatitis C testing and treatment into routine HIV care in Cameroon is feasible

**DOI:** 10.1002/jia2.26417

**Published:** 2025-02-12

**Authors:** Mathurin Pierre Kowo, Liza Coyer, Victor Sini, Carole Assontsa Kafack, Gabriella Yelheen Metomo, Guy S. Wafeu, Richard Njouom, Alexander Boers, Roel Coutinho, Oudou Njoya, Charles Kouanfack, the DHEPC project team

**Affiliations:** ^1^ Research Laboratory on Viral Hepatitis and Health Communication University of Yaoundé I Yaoundé Cameroon; ^2^ ECDC Fellowship Programme, Field Epidemiology path (EPIET), European Centre for Disease Prevention and Control (ECDC) Stockholm Sweden; ^3^ State Institute for Health II, Task Force for Infectious Diseases (GI), Bavarian Health and Food Safety Authority (LGL) Munich Germany; ^4^ HIV/AIDS Approved Treatment Center Yaounde General Hospital Yaounde Cameroon; ^5^ Department of Clinical Sciences Higher Institute of Medical Technology of Nkolodom Yaounde Cameroon; ^6^ Faculty of Medicine and Pharmaceutical Sciences University of Dschang Dschang Cameroon; ^7^ French National Agency for Research on AIDS and Infectious Diseases, Cameroon Site, Central Hospital of Yaoundé Yaoundé Cameroon; ^8^ PharmAccess Foundation Amsterdam the Netherlands; ^9^ Centre Pasteur of Cameroon Yaoundé Cameroon; ^10^ Joep Lange Institute Amsterdam the Netherlands; ^11^ Centre for Research of Emergency and Re‐emergency Diseases Yaoundé Cameroon

**Keywords:** Cameroon, diagnosis, direct‐acting antiviral, hepatitis C virus, HIV, implementation sciences

## Abstract

**Introduction:**

Hepatitis C virus (HCV) prevalence and adverse outcomes are higher among people with human immunodeficiency virus (HIV) than people without HIV. Yet, HCV prevalence among people with HIV in Cameroon remains unknown, with HCV diagnosis and treatment largely inaccessible due to care centralization by specialists with high out‐of‐pocket costs. Integration of HCV services into routine HIV care by general practitioners could improve diagnosis and treatment coverage. We aimed to examine HCV prevalence and treatment cure rate among people with HIV attending 11 HIV clinics in the Centre Region of Cameroon.

**Methods:**

We offered HCV rapid antibody testing, and, if positive, RNA testing to all persons ≥21 years, on HIV ART for ≥6 months and with suppressed HIV RNA (<1000 copies) who attended HIV counselling and treatment appointments between 20 April 2021 and 31 May 2022. Participants with an HCV RNA positive test received 12 weeks of pangenotypic sofosbuvir/velpatasvir. We calculated the cure rate as the proportion of participants with a sustained virological response 12 weeks after treatment completion (SVR12) among all starting and completing treatment.

**Results:**

We tested 8266 persons for HCV antibodies, 316 (3.8%, 95% CI = 3.4−4.3%) of whom were anti‐HCV positive. Of these, 286 (90.5%) were sampled for HCV RNA, 20 (6.3%) ineligible, 5 (1.6%) declined, 4 (1.3%) left before sampling and 1 (0.3%) had an unknown reason. Among 286 sampled, 251 (87.8%) had detectable HCV RNA. Of these, 173 (68.9%) enrolled for treatment, 55 (21.9%) were eligible but not enrolled (49 lost‐to‐follow‐up, 6 denied) and 23 (9.2%) were ineligible. Of 173 enrolled, 165 completed treatment, 6 were lost‐to‐follow‐up and 2 were excluded due to treatment interruption. SVR12 was achieved in 93.6% (*n* = 162; 95% CI: 88.9–96.8%) of those enrolled and 98.2% (95% CI: 94.8–99.6%) of treatment completers. All three initially not achieving SVR12 were cured with second‐line treatment (sofosbuvir/velpatasvir/voxilaprevir).

**Conclusions:**

Our study demonstrates the viability of integrating HCV testing and treatment into routine HIV care in Cameroon, yielding new HCV diagnoses and high cure rates. Cameroon can use this strategy to achieve HCV elimination goals, although improvements in testing uptake, diagnosis and treatment access, and laboratory capacity are needed.

## 1 INTRODUCTION

Hepatitis C virus (HCV) infection is a major cause of liver cirrhosis, hepatocellular carcinoma and liver failure, resulting in 15.3 million disability‐adjusted life years and 540,000 deaths globally in 2019 [1, 2]. The introduction of direct‐acting antivirals (DAAs) has greatly reduced newly reported HCV diagnoses, prevalence and HCV‐related mortality, also among people with human immunodeficiency virus (HIV), especially in high‐access settings [3, 4, 5]. HCV cure rates with DAA treatment are very high (>90%), also in persons with HCV/HIV co‐infection [6, 7, 8, 9] and for pangenotypic regimens [10, 11, 12]. However, low‐ and middle‐income countries still face challenges in accessing testing and treatment, hindering progress in HCV diagnosis and treatment coverage [13, 14]. The WHO African region accounted for 9.2% of the globally estimated 57.8 million people with chronic HCV infection in 2019 [4]. Cameroon, a lower‐middle‐income country in Central Africa with 27 million inhabitants, faces a high HCV prevalence, estimating 190,000 people with HCV, but few receive diagnosis and treatment due to resource constraints and inadequate healthcare infrastructure [15, 16, 17].

HCV prevalence and adverse outcomes are higher among people with HIV than people without HIV [18, 19, 20]. In Cameroon, approximately 480,000 people have HIV [21], with 88% on antiretroviral treatment (ART) in 2022. Limited studies estimate HCV seroprevalence among Cameroonians with HIV from 5% to 38% and viraemia prevalence from 8% to 21% [22]. Integrating HCV testing and treatment within existing HIV care structures could improve diagnosis and treatment coverage. Nonetheless, no studies have evaluated the feasibility and outcomes of implementing such an approach within this context.

Previously, two demonstration projects in Yaoundé showed high cure rates exceeding 95% after DAA treatment provided by gastroenterologists to people with HCV identified through clinics and blood banks [23, 24]. This third study, Decentralisation of Hepatitis C (DHEPC), aimed to assess the feasibility of integrating HCV testing and pan‐genotypic DAA treatment into routine HIV care provided by general practitioners (GPs) in 11 HIV clinics in the Centre region of Cameroon.

## 2 METHODS

### 2.1 Study design and population

DHEPC was a prospective, longitudinal demonstration study among people with HIV in 11 HIV clinics in Cameroon's Centre region, approved by the Cameroon National Ethics Committee (N°2020/07/1271/CE/CNERSH/SP, N°2021/10/1389/CE/CNERSH/SP) and the Ministry of Health (631‐31‐20). All participants provided written informed consent.

### 2.2 HCV rapid antibody testing and treatment

The study recruited people for HCV rapid antibody testing from all persons attending routine HIV counselling and treatment visits between 20 April 2021 and 31 May 2022. Eligibility criteria for HCV antibody testing included age ≥21 years (i.e. the legal age in Cameroon), ≥6 months on HIV ART, suppressed HIV ribonucleic acid (RNA) (<1000 copies) dating ≤6 months and clinical stability.

The eligibility criterion for HCV treatment was testing anti‐HCV and HCV RNA positive. Exclusion criteria included hepatitis B virus (HBV) surface antigen positivity, current HCV DAA treatment, renal impairment (creatinine clearance <50 ml/minute), a history of decompensated cirrhosis, hepatocellular carcinoma, liver or kidney transplantation and current use of amiodarone, carbamazepine, phenytoin, phenobarbital, oxcarbazepine, rifabutin, rifampin, rifapentine, St. John's wort or rosuvastatin. Pregnant persons or those anticipating pregnancy, persons with serious medical or psychiatric conditions affecting treatment or adherence and persons unable to attend follow‐up visits were also excluded. Persons receiving an HIV ART regimen including efavirenz or nevirapine were switched to atazanavir/ritonavir or dolutegravir before starting treatment.

HCV treatment consisted of a 12‐week course of generic sofosbuvir/velpatasvir (Mylan, 400/100 mg). Participants not achieving sustained virological response 12 weeks after treatment completion (SVR12) were offered a 12‐week course of branded sofosbuvir/velpatasvir/voxilaprevir (Gilead) as second‐line treatment. GPs provided treatment, supervised by gastroenterologists.

### 2.3 Procedures

HCV rapid antibody testing was offered during routine HIV care visits, including individual appointments and group counselling sessions. Study physicians or nurses explained the objectives, procedures, potential benefits and risks, addressing any questions. Interested persons received further details and were assessed for eligibility. Eligible persons provided written informed consent before testing. After consent, participants underwent HCV rapid antibody testing, and those testing anti‐HCV positive were immediately invited to proceed with pre‐enrolment.

Pre‐enrolment involved verifying eligibility through patient history, routine HIV lab results, a physical exam and pre‐inclusion blood samples for further tests. Eligibility was reconfirmed at enrolment before starting treatment. Persons ineligible for our study, such as those with hepatitis B infection or kidney failure, were referred to higher levels of care.

Enrolment and follow‐up visits occurred at weeks 0, 4, 8, 12, 24 and 26, and were coordinated with routine HIV ART treatment pick‐ups. This arrangement minimized additional visits, requiring only one extra visit beyond routine HIV care to one to receiving the HCV RNA result. At each visit, participants received a physical exam and counselling on HIV/HCV treatment adherence, family planning and HCV reinfection prevention. They also received a 4‐week treatment supply from the on‐site pharmacy. At weeks 8 and 24, blood samples were collected for laboratory testing. Those who did not achieve SVR12 had a follow‐up blood test, if necessary, were invited for second‐line treatment.

### 2.4 Laboratory investigations

Laboratory testing followed standard HIV care, including annual HIV RNA testing and 6‐monthly creatinine tests for those on tenofovir‐based ART. Participants without a recent HIV RNA test (≤6 months) were tested before enrolment.

HCV antibody testing used the Hexagone HCV rapid test [20]. If positive, further testing included HCV RNA (GeneExpert), hepatitis B surface antigen (HBsAg), creatinine, alanine aminotransferase, aspartate aminotransferase, full blood counts, and, for those an APRI score suggestive of cirrhosis, albumin and bilirubin. Women of childbearing age had urine β‐hCG tests at pre‐enrolment, enrolment, and weeks 4 and 8. HCV RNA was retested at week 24.

Most tests were performed on site (if possible); however, HCV RNA and HBsAg were conducted by the Centre Pasteur of Cameroon (CPC). CPC also served as the reference laboratory for routine HIV investigations, including RNA testing for five of the 11 participating clinics, and performed other tests if on‐site testing faced capacity or quality issues.

### 2.5 Financial contribution

As per Ministerial decision D13‐124/MINSANTE/DLM/SD/VIH‐SIDA‐ISTANBUL, the price for 28 tablets of sofosbuvir/velpatasvir (400/100 mg), covering 4 weeks of standard HCV treatment in Cameroon, was set at 100,000 Central African CFA Franc (XAF) on 20 July 2019. In routine care, a 12‐week course of treatment, plus pre‐treatment laboratory tests at 400,000 XAF and follow‐up tests at 150,000 XAF, would amount to a total treatment cost of 850,000 XAF (around €1295 or United States Dollar $1420). Participants in this study were asked to pay a treatment contribution of 50,000 XAF (around €76 or $84). If participants accepted treatment but were unable to pay, the principal investigator and site physician could offer subsidized or free‐of‐charge participation. Travel expenses for enrolled participants were reimbursed.

### 2.6 Data collection

Electronic data collection included demographics, clinical information, financial hardship (using the 10‐item Poverty Probability Index [PPI] questionnaire to estimate the probability of living below the national poverty line [25]) and HCV treatment adherence assessment (using the 6‐item Test for Evaluating Observance questionnaire where 0 = good, 1–2 = suboptimal, 3 ≥ insufficient [26]).

### 2.7 Outcome measures

Primary outcomes were HCV seroprevalence, detectable HCV RNA and post‐treatment SVR12. Secondary outcomes included treatment initiation rates, lead times from HCV antibody testing to treatment enrolment, ability to pay a contribution of 50,000 XAF, adherence and adverse effects.

### 2.8 Statistical analysis

Statistical analysis comprised prevalence and cure rates with 95% confidence intervals (CI) using the exact method and comparisons of demographic and clinical factors using Pearson's chi‐square, Wilcoxon rank‐sum or Fisher's exact tests as appropriate. R version 4.0.2 (Vienna, Austria) was used for data cleaning and analysis.

## 3 RESULTS

### 3.1 HCV rapid antibody testing

We tested 8266 persons for HCV antibodies, who had a median age of 47.4 years (interquartile range [IQR] = 39.7−55.7), 6204 (75.7%) were female, and 564 (6.9%) had one or more comorbidities, primarily hypertension (Table 1). Three hundred and sixteen (3.8%, 95% CI = 3.4−4.3%) tested anti‐HCV positive. Compared to participants testing anti‐HCV negative, participants testing positive were older, more often had comorbidities, and were more likely to report any risk factor for HCV transmission and severe disease.

**Table 1:** Descriptive characteristics and risk factors for HCV transmission and severe disease, overall by HCV rapid antibody test result (*N* = 8266), NoCo study, April 2021−May 2022, Centre Region of Cameroon

Characteristic or factor	Total tested, *N* = 8266 (100%)	Positive, *N* = 316 (3.8%)	Negative, *N* = 7950 (96.2%)	*p*‐value
Age (median, IQR)	47.4 (39.7, 55.7)	58.6 (49.5, 65.2)	47.0 (39.4, 55.1)	<0.001
Missing	75	0	75	
**Age category**				<0.001
20−39	2128 (26.0%)	29 (1.4%)	2099 (98.6%)	
40−59	4794 (58.5%)	146 (3.0%)	4648 (97.0%)	
60−79	1260 (15.4%)	139 (11.0%)	1121 (89.0%)	
80−94	11 (0.1%)	2 (18.2%)	9 (81.8%)	
Missing	73	0	73	
**Sex**				0.5
Female	6204 (75.8%)	245 (3.9%)	5959 (96.1%)	
Male	1986 (24.2%)	71 (3.6%)	1915 (96.4%)	
Missing	76	0	76	
**Civil status** ^a^				<0.001
Married	2375 (29.2%)	85 (3.6%)	2290 (96.4%)	
Widowed	1440 (17.7%)	113 (7.8%)	1327 (92.2%)	
Single	2844 (34.9%)	88 (3.1%)	2756 (96.9%)	
Divorced	168 (2.1%)	7 (4.2%)	161 (95.8%)	
Cohabiting	1317 (16.2%)	23 (1.7%)	1294 (98.3%)	
Missing	122	0	122	
**Employment status** ^a^				<0.001
Civil servant	664 (8.2%)	15 (2.3%)	649 (97.7%)	
Employed in private sector	726 (8.9%)	13 (1.8%)	713 (98.2%)	
Self‐employed	4381 (53.8%)	112 (2.6%)	4269 (97.4%)	
Unemployed	2225 (27.3%)	166 (7.5%)	2059 (92.5%)	
Other	148 (1.8%)	10 (6.8%)	138 (93.2%)	
Missing	122	0	122	
**Any comorbidities** ^a^				<0.001
No	7574 (93.1%)	252 (3.3%)	7322 (96.7%)	
Yes	564 (6.9%)	63 (11.2%)	501 (88.8%)	
Missing	128	1	127	
**Chronic HBV** ^a^				0.3
No	8114 (99.7%)	314 (3.9%)	7800 (96.1%)	
Yes	25 (0.3%)	2 (8.0%)	23 (92.0%)	
Missing	127	0	127	
**Diabetes** ^a^				<0.001
No	8030 (98.7%)	296 (3.7%)	7734 (96.3%)	
Yes	109 (1.3%)	20 (18.3%)	89 (81.7%)	
Missing	127	0	127	
**Hypertension** ^a^, ^c^				<0.001
No	7744 (95.1%)	270 (3.5%)	7474 (96.5%)	
Yes	395 (4.9%)	46 (11.6%)	349 (88.4%)	
Missing	127	0	127	
**Poor mental health** ^a^, ^d^				>0.9
No	8130 (99.9%)	316 (3.9%)	7814 (96.1%)	
Yes	9 (0.1%)	0 (0%)	9 (100%)	
Missing	127	0	127	
**Other comorbidities than HBV, diabetes, hypertension and poor mental health** ^a^				<0.001
No	8041 (98.8%)	303 (3.8%)	7738 (96.2%)	
Yes	98 (1.2%)	13.3 (13%)	85 (86.7%)	
Missing	127	0	127	
**Comedication (other than ART)** ^a^				<0.001
No	7064 (87.2%)	217 (3.1%)	6847 (96.9%)	
Yes	1037 (12.8%)	97 (9.4%)	940 (90.6%)	
Missing	165	2	163	
**Traditional medication** ^a^				<0.001
No	7731 (95.1%)	265 (3.4%)	7466 (6.67%)	
Yes	397 (4.9%)	50 (12.6%)	347 (87.4%)	
Missing	138	1	137	
**Blood transfusion** ^d^				<0.001
No	7193 (88.5%)	244 (3.4%)	6949 (96.6%)	
Yes	939 (11.5%)	72 (7.7%)	867 (92.2%)	
Missing	134	0	134	
**Surgery** ^d^				<0.001
No	6742 (82.9%)	226 (3.4%)	6516 (96.6%)	
Yes	1390 (17.1%)	90 (6.5%)	1300 (93.5%)	
Missing	134	0	134	
**Scarring or tattoos** ^a^				0.014
No	6746 (82.9%)	246 (3.6%)	6500 (96.4%)	
Yes	1388 (17.1%)	70 (5.0%)	1318 (95.0%)	
Missing	132	0	132	
**History of collective vaccination** ^b^, ^e^				<0.001
No	6554 (81.1%)	225 (3.4%)	6329 (96.6%)	
Yes	1527 (18.9%)	89 (5.8%)	1438 (94.2%)	
Missing	185	2	183	
**Injecting drug use** ^d^				>0.9
No	8122 (99.9%)	315 (3.9%)	7807 (96.1%)	
Yes	5 (<0.1%)	0 (0%)	5 (100%)	
Missing	139	1	138	
**Contact with people living with HCV in daily life** ^a^				0.007
No	8034 (98.9%)	304 (3.8%)	7730 (96.2%)	
Yes	88 (1.1%)	9 (10.2%)	79 (89.8%)	
Missing	144	3	141	
**Consumes alcohol** ^a^				0.025
No	4160 (51.1%)	142 (3.4%)	4018 (96.6%)	
Yes	3979 (48.9%)	174 (4.4%)	3805 (95.6%)	
<13 units per week	2125 (53.4%)	77 (3.6%)	2048 (96.4%)	
≥14 units per week	1854 (46.6%)	97 (5.2%)	1757 (94.8%)	
Missing	127	0	127	

Abbreviations: ART, antiretroviral therapy; HBV, hepatitis B virus; HCV, hepatitis C virus; IQR, interquartile range.

^a^ Current.

^b^ Systolic blood pressure >140 mmHg, diastolic > 9 mmHg, or if the participant was taking medication for hypertension.

^c^ Defined at the discretion of the study physician or nurses based on the participant's statements.

^d^ Ever.

^e^ HCV prevalence in Cameroon greatly varies by age and region, a disparity purportedly attributed to historic mass treatment and vaccination campaigns utilizing unsterilized needles and syringes [16, 17].

Of 316 participants testing anti‐HCV positive, 286 (90.5%) were sampled for HCV RNA, 20 (6.3%) were ineligible, 5 (1.6%) declined, 4 (1.3%) left before sampling and 1 (0.3%) had an unknown reason. Of 286 sampled, 251 (87.8%) had detectable HCV RNA.

### 3.2 HCV treatment

**Figure 1:**
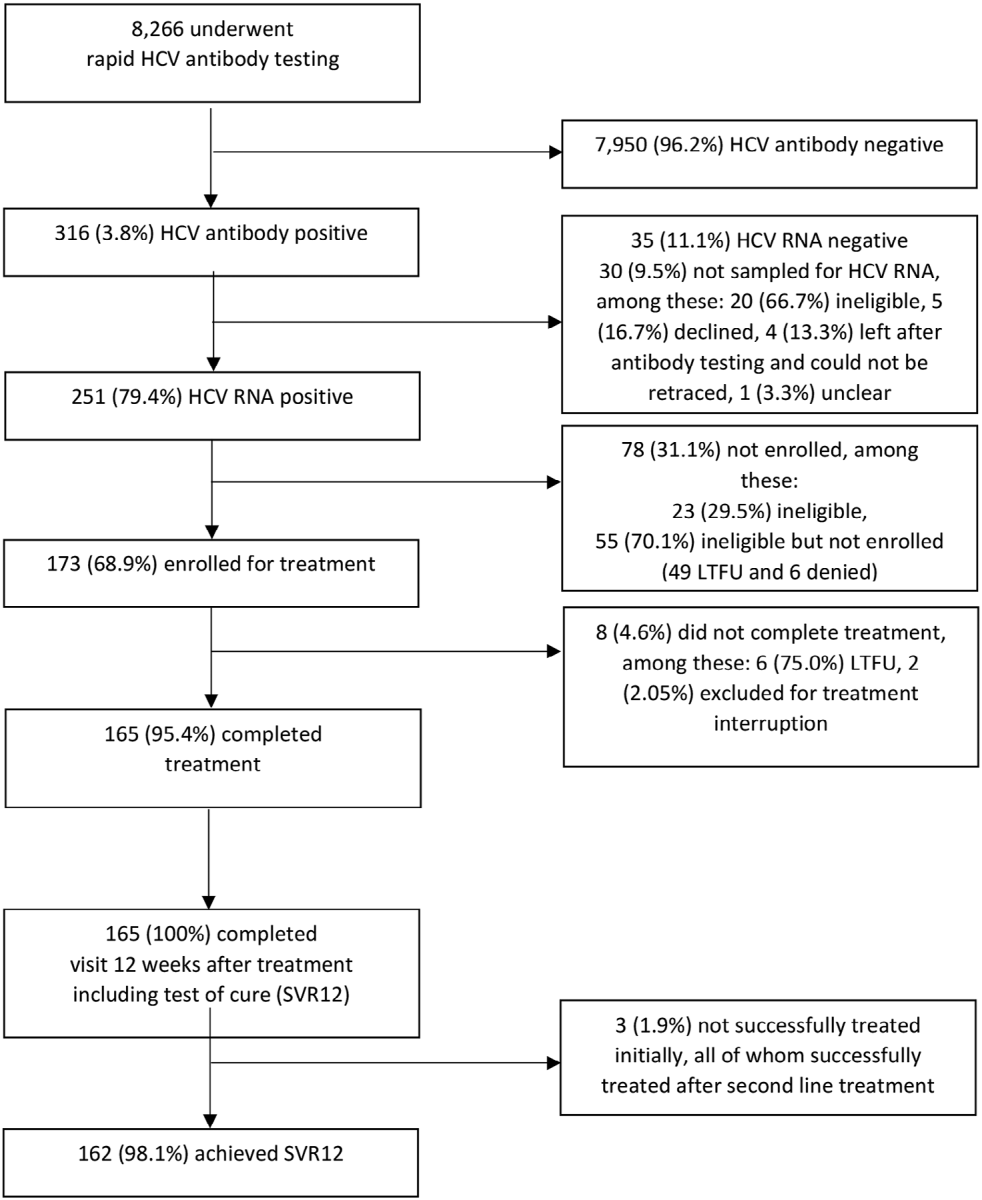
Flow chart of HCV rapid antibody testing, enrolment and treatment, NoCo study, April 2021−March 2023, Centre Region of Cameroon. Abbreviation: LTFU, lost to follow‐up.

Of 251 with detectable HCV RNA, 173 (68.9%) enrolled for treatment, 55 (21.9%) were eligible but not enrolled (49 lost‐to‐follow‐up and 6 denied) and 23 (9.2%) were ineligible (Figure 1). The median time from pre‐inclusion laboratory results to enrolment was 5.0 weeks (IQR = 2.1−13.5), varying from 2.4 to 18.5 weeks between sites. Enrolled participants had a median age of 58.9 years (IQR = 51.4−64.9), 137 (79.1%) were female and 38 (21.9%) had one or more comorbidities, primarily hypertension (Table 2). Their reported primary risk factor for HCV transmission was collective vaccination (31.9%), followed by surgery (28.9%). Their median PPI score was 52.0 (IQR = 34.0−65.0), while 152 (87.9%) fully or partially paid the treatment contribution.

**Table 2:** Descriptive characteristics and risk factors for HCV transmission and severe disease, among those enrolled for HCV treatment (*N* = 173), NoCo study, April 2021−May 2022, Centre Region of Cameroon

Characteristic or factor	Total enrolled, *N* = 173 (100%)
**Age (median, IQR)**	58.9 (51.4, 64.9)
**Age category**	
20−39	12 (6.9%)
40−59	81 (46.8%)
60−79	79 (45.7%)
80−94	1 (0.6%)
**Sex**	
Female	137 (79.2%)
Male	36 (20.8%)
**Civil status** ^a^	
Married	53 (30.6%)
Widowed	61 (35.3%)
Single	47 (27.2%)
Divorced	4 (2.3%)
Cohabiting	8 (4.6%)
**Employment status** ^a^	
Civil servant	9 (5.2%)
Employed in private sector	6 (3.5%)
Self‐employed	63 (36.4%)
Unemployed	87 (50.3%)
Other	8 (4.6%)
**PPI (median, IQR)** ^a^	52.0 (34.0, 65.0)
**Any comorbidities** ^a^	
No	135 (78.0%)
Yes	38 (22.0%)
**Chronic HBV** ^a^	
No	173 (100%)
Yes	0 (0%)
**Diabetes** ^a^	
No	160 (92.5%)
Yes	13 (7.5%)
**Hypertension** ^a^, ^c^	
No	144 (83.2%)
Yes	29 (16.8%)
**Poor mental health** ^a^, ^d^	
No	173 (100%)
Yes	0 (0%)
**Other comorbidities than HBV, diabetes, hypertension and poor mental health** ^a^	
No	168 (97.1%)
Yes	5 (2.9%)
**Comedication (other than ART)** ^a^	
No	115 (66.5%)
Yes	58 (33.5%)
**Traditional medication** ^a^	
No	152 (88.4%)
Yes	20 (11.6%)
Missing	1
**Blood transfusion** ^d^	
No	130 (75.1%)
Yes	43 (24.9%)
**Surgery** ^d^	
No	123 (71.1%)
Yes	50 (28.9%)
**Scarring or tattoos** ^a^	
No	140 (80.9%)
Yes	33 (19.1%)
**History of collective vaccination** ^b^, ^e^	
No	117 (68.0%)
Yes	55 (32.0%)
Missing	1
**Injecting drug use** ^d^	
No	172 (100%)
Yes	0 (0%)
Missing	1
**Contact with people living with HCV in daily life** ^a^	
No	164 (95.9%)
Yes	7 (4.1%)
Missing	2
**Consumes alcohol** ^a^	
No	78 (45.1%)
Yes	95 (54.9%)
<13 units per week	41 (43.2%)
≥14 units per week	54 (56.8%)
Missing	78

Abbreviations: ART, antiretroviral therapy; HBV, hepatitis B virus; HCV, hepatitis C virus; IQR, interquartile range.

^a^ Current.

^b^ Systolic blood pressure >140 mmHg, diastolic > 9 mmHg, or if the participant was taking medication for hypertension.

^c^ Defined at the discretion of the study physician or nurses based on the participant's statements.

^d^ Ever.

^e^ HCV prevalence in Cameroon greatly varies by age and region, a disparity purportedly attributed to historic mass treatment and vaccination campaigns utilizing unsterilized needles and syringes [16, 17].

Among 173 enrolled for treatment, 165 (95.4%) completed treatment, 6 (3.5%) were lost‐to‐follow‐up and 2 (1.2%) were excluded due to treatment interruption (Figure 1). SVR12 was achieved in 93.6% (*n* = 162; 95% CI: 88.9–96.8%) of those enrolled and 98.2% (95% CI: 94.8–99.6%) of treatment completers. All three initially not achieving SVR12 were cured with second‐line treatment. Overall, the percentage with good treatment adherence was high throughout follow‐up, with 87.1% at week 4, 88.2% at week 8 and 93.4% at week 12. Adverse events reported were rare and mostly restricted to nausea (*n* = 18, 10.4%), asthenia (*n* = 13, 7.5%), headache (*n* = 13, 7.5%), diarrhoea (*n* = 6, 3.5%) and muscle aches (*n* = 5, 2.9%) usually at treatment onset.

## 4 DISCUSSION

In this analysis of individuals with HIV in Cameroon, we found an HCV seroprevalence of 3.8%, with 87.8% exhibiting active viraemia, and a high HCV cure rate of 98.2% with pan‐genotypic DAA treatment administered by GPs at HIV clinics. Rapid HCV antibody testing integrated into the HIV treatment cascade enabled the identification and treatment of otherwise undetected infections. The seroprevalence exceeded smaller previous studies in Cameroon [22], the global estimate of 2.4% across 30 general population samples of people with HIV [27] and the 2.5% prevalence from the 2011 Demographic and Health Survey [16]. Furthermore, the high prevalence of active viraemia among those with antibodies suggests a combined prevalence greater than the estimated 0.7% viraemia prevalence for Cameroon in 2020, which included individuals without HIV [14]. These differences could reflect a higher prevalence of transmission risk factors and limited access to prevention and treatment in our setting compared to other settings, with a history of collective vaccination identified as the most common risk factor, consistent with the historical epidemiology of hepatitis C in Cameroon [16, 17].

The high cure rate corroborates previous studies in Yaoundé [23, 24] and international findings for sofosbuvir/velpatasvir in people with HIV [10]. This rate was achieved with high treatment adherence, minimal adverse events and few additional study visits. It demonstrates the feasibility of decentralizing HCV care to non‐specialist settings, with GPs effectively managing HCV care using standardized protocols and specialist supervision. GPs are more accessible compared to specialists due to their familiarity with and proximity to patients and lower travel and consultation time and costs. Furthermore, it underscores the effectiveness of pan‐genotypic DAA treatment in individuals with HIV/HCV coinfection, eliminating the need for costly and time‐consuming HCV genotyping and individualized treatment.

Our findings provide evidence for the development of national guidelines for HCV treatment and care in individuals with HIV in Cameroon, emphasizing the need for affordable point‐of‐care diagnostics, simplified diagnostic and treatment algorithms, minimal number of visits and seamless integration into existing healthcare frameworks. However, some laboratories faced technical limitations in diagnostic performance and sample storage, which were mitigated by a courier system of motorbike riders to transport samples to the CPC. Sustainable implementation will require improving laboratory capacity, personnel training and quality assurance alongside decentralization.

Despite cost reductions, high treatment costs still hinder access. While most participants could pay the study's treatment contribution, 13% were unable to do so despite the already heavily subsidized costs compared to routine care, highlighting the need for alternative financing mechanisms to ensure broader access to HCV treatment for people with HIV/HCV coinfection.

We tested 8266 individuals for HCV antibodies, falling short of our 20,000 target. We did not collect data on those who declined testing, limiting our ability to assess uptake and compare characteristics between tested and untested individuals. Several factors may explain the low testing numbers. Firstly, the acceptability of rapid antibody testing among people with HIV might have been limited. Secondly, the workload of participating healthcare providers may have impacted recruitment efforts. Some sites used group counselling sessions instead of individual sessions for recruitment, relying more on active requests. Thirdly, our estimate of potential study candidates may have been overestimated. Many listed as receiving care from participating clinics may not have attended during the study period, potentially due to community HIV medication distribution or COVID‐19 concerns. Additionally, 31% of participants were lost‐to‐follow‐up between RNA testing and enrolment, possibly due to long distances to clinics, insufficient transportation funds (despite reimbursement), time constraints or low awareness of treatment needs.

## 5 CONCLUSIONS

Our study demonstrates the feasibility of integrating HCV rapid antibody testing and treatment into routine HIV care by GPs in Cameroon, resulting in high cure rates. However, there is room for improvement in on‐site laboratory capacity and acceptance of testing, along with better access to treatment, to ensure successful routine implementation.

## COMPETING INTERESTS

The authors report no conflicts of interest.

## AUTHORS’ CONTRIBUTIONS

AB, CK, ON and RC designed the study and wrote the study protocol. AB, CAK, CK, GYM, MPK, ON, RN, RC and VS contributed to the implementation of the study, through coordination, inclusion and follow‐up, supervision or laboratory testing. GSW designed the database. CAK, GSW and LC verified the data. LC analysed the data and wrote and revised the manuscript, under the supervision of RC. All authors approved the final version of the manuscript for publication.

## FUNDING

Gilead Sciences fully funded the DHEPC project as Investigator Sponsored Research. Gilead Sciences had no influence on the study design, data collection, data analysis, or interpretation of the data, in the writing of the manuscript, or in the decision to publish results. Treatment medication was funded separately through the PharmAccess Foundation and the Joep Lange Institute.

## Data Availability

The data that support the findings of this study are available on request from the corresponding author. The data are not publicly available due to privacy or ethical restrictions.
